# The mediating effect of e-health literacy on social support and behavioral decision-making on glycemic management in pregnant women with gestational diabetes: a cross-sectional study

**DOI:** 10.3389/fpubh.2024.1416620

**Published:** 2024-07-17

**Authors:** Peng Yumei, Ke Huiying, Shen Liqin, Zhao Xiaoshan, Zhao Meijing, Xie Yaping, Zhao Huifen

**Affiliations:** ^1^School of Nursing, Fujian Medical University, Fuzhou, China; ^2^Clinical Nursing Teaching and Research Department, Second Affiliated Hospital of Fujian Medical University, Quanzhou, Fujian, China; ^3^Gynaecology and Obstetrics Department, Second Affiliated Hospital of Fujian Medical University, Quanzhou, Fujian, China

**Keywords:** gestational diabetes, pregnancy, glycemic management, behavioral decision making, health behavior, social support

## Abstract

**Background:**

Social support and e-health literacy are closely related to individual health behaviors, while behavior is premised on decision-making. Few studies have identified the relationships among social support, e-health literacy, and behavioral decision-making, and the nature of these relationships among pregnant women with gestational diabetes remains unclear. Therefore, this study aimed to investigate relationships among social support, e-health literacy, and glycemic management behavioral decisions in pregnant women with gestational diabetes.

**Methods:**

Using continuous sampling, an online cross-sectional survey was conducted among pregnant women with gestational diabetes who met the inclusion and exclusion criteria at four Class 3 hospitals in Fujian Province from October to December 2023. A structured questionnaire was used to collect data on general characteristics, socioeconomic status, social support, e-health literacy, and behavioral decision-making regarding glycemic management. Descriptive statistical analyses, correlation analyses, and mediation effects were used to assess associations.

**Results:**

A total of 219 pregnant women with gestational diabetes participated, and 217 valid results were obtained. The level of glycemic management behavior decision-making in women with gestational diabetes was positively correlated with e-health literacy (*r* = 0.741, *p* < 0.01) and with perceived social support (*r* = 0.755, *p* < 0.01). E-health literacy was positively correlated with perceived social support (*r* = 0.694, *p* < 0.01). The indirect effect of perceived social support on glycemic management behavior decisions through e-health literacy (a*b) was 0.153, accounting for 38% of the total effect.

**Conclusion:**

Social support and e-health literacy in pregnant women with gestational diabetes are related to behavioral decision-making in glycemic management. The results of this study provide a reference for developing targeted measures to improve glycemic management behaviors in pregnant women with gestational diabetes, which is crucial for achieving sustainable glycemic management.

## 1 Introduction

Gestational Diabetes Mellitus (GDM) is the most common metabolic disease during pregnancy and is defined as an abnormal glucose tolerance that first occurs or is discovered during pregnancy (1). The Global Diabetes Map data report from the International Diabetes Federation points out (2) that 16.2% of pregnant women worldwide experience varying degrees of elevated blood sugar, of which 86.4% are caused by GDM. In China, the overall incidence of GDM is 14.8% and the number of pregnant women with GDM ranks among the highest worldwide (3). With the increasing prevalence of unhealthy lifestyles and the development of assisted reproductive technology, the number of GDM cases will continue to increase. The high incidence of GDM poses a huge burden on the social economy (4). In 2017, the global average additional cost for patients with gestational diabetes was US$15,593, an increase of 8% from the 2015 statistics (5). The average cost of diagnosis and treatment for a Chinese pregnant woman with GDM is US$6,677.37, which is 1.95 times higher than that for a normal pregnant woman. The annual social and economic burden is approximately US$19.36 billion (6).

More importantly, GDM is the most direct risk factor for short- and long-term adverse health outcomes in pregnant women and their offspring. GDM not only directly leads to an increase in the incidence of adverse health outcomes for pregnant women and their offspring but also leads to a significant increase in the incidence of depression and other negative emotional reactions. First, pregnant women with GDM have a significantly increased risk of developing glucose metabolism disorders and type 2 diabetes mellitus (T2DM) in the future. Studies have shown that 50% of women with a history of GDM have an abnormal glucose metabolism rate of 42.2% within 2 years of delivery, an incidence rate of glucose impairment of 18.4%, a cumulative incidence rate of diabetes of 17.4%, and impaired glucose metabolism that persists for up to 10 years (7, 8). Daly et al. (9) conducted a retrospective cohort study and showed that pregnant women with GDM have a significantly higher risk of developing hypertension and T2DM than pregnant women with normal blood sugar levels. Second, as the mother’s blood sugar levels increase, the chances of adverse pregnancy outcomes such as hypoglycemia, macrosomia, and intrauterine distress also increase (10, 11). A study from Finland also showed that the offspring of patients with GDM have a higher risk of congenital malformations (12). GDM can also cause psychological distress to the mother, which, in turn, affects the normal growth and development of the fetus. In a prospective longitudinal study, Fraser et al. (13) found that the anxiety and depression scores of women with GDM who used insulin or who controlled their blood sugar through diet were significantly higher than those of women without GDM. Riggin et al. (14) also found that GDM is associated with severe mental illness. A meta-analysis by Rowland et al. (15) showed that maternal exposure to gestational diabetes was associated with autism spectrum disorder. Therefore, preventing, controlling, and reducing the incidence of GDM and its individual and social burdens are tasks that cannot be ignored in the global healthcare of pregnant women.

Glycemic management in pregnant women with GDM is considered the most cost-effective prevention and control strategy (16, 17). However, existing studies (18–21) indicate that the glycemic management level of pregnant women with GDM is moderate to low. To this end, many scholars (22–24) have conducted empirical intervention studies to improve blood sugar management in pregnant women with GDM. Although it is very effective in promoting individual management behavior changes in the short term, many challenges remain in changing lifestyles and maintaining blood sugar management behaviors in pregnant women with GDM. From a behavioral science perspective (25), the process of individual behavior change is closely related to behavioral decision making. The premise of action is decision making, which involves individual cognition, ability, and the external environment. In addition, as a special vulnerable group, social support can greatly affect blood sugar management decision-making behavior in pregnant women with GDM (26). With the development of information technology, the status of traditional medical staff as the main transmitters of disease-related information has changed. The frequency with which pregnant women with GDM obtain health information through electronic devices has also increased. The ability to seek and obtain information to understand their diseases are referred to as electronic health (e-health) literacy, which has a critical effect on glycemic management decisions (27, 28). Some studies (29, 30) have pointed out that in the digital environment, e-health literacy is a key factor affecting health because it can mobilize individuals’ enthusiasm to actively participate in health management and improve individual motivation for health behaviors. The comprehensive model of information acquisition (31, 32) proposes that in the digital environment, complete social support can enhance people’s information management effectiveness and improve e-health literacy. However, previous e-health-related research focused on online public health and e-health intervention measures (33). The target groups focused on older adults, students, and some people with chronic diseases. Few studies have been conducted on the e-health literacy of pregnant women with GDM. The relationship and influence paths among social support, e-health literacy, and glycemic management behavioral decision-making in pregnant women with GDM have not yet been reported. Considering this, this study explored the relationship between social support and behavioral decision-making regarding blood sugar management in pregnant women with GDM, as well as the factors mediating this association. We proposed the following hypotheses: (1) Social support is positively related to behavioral decision-making regarding glycemic management. (2) E-health is positively correlated with behavioral decision-making regarding glycemic management. (3) E-health literacy mediates the effects of social support and behavioral decisions on glycemic management.

## 2 Materials and methods

### 2.1 Participants

Using a continuous sampling method, pregnant women with GDM who met the admission and discharge standards at four tertiary hospitals in Fujian Province were selected for investigation from October 2023 to December 2023. The following inclusion criteria were used: (1) diagnosis of GDM by a 75 g oral glucose tolerance test (16); (2) regular prenatal check-up in the obstetrics clinic; (3) good Chinese language expression and communication skills; and (4) informed consent and voluntary participation in this study. Patients with severe pregnancy-related complications or comorbidities were excluded (34). The following sample size formula was used (35): *N* = [number of variables × (5–10) × [1 + (10–15%)]]. There were variables in this survey and considering that 10% of the questionnaires were invalid, at least 189 participants were included. The final sample size was 217.

### 2.2 Measures

#### 2.2.1 Evaluation of related variables

This study included general information, such as age, gestational period, place of residence, education level, family history of diabetes, occupation, current method of controlling blood sugar, history of antidiabetic drug use, type of medical insurance, and *per capita* monthly household income.

#### 2.2.2 Evaluation of the dependent variables

The Behavioral Decision-making scale for glycemic management in pregnant women with GDM was developed by the researchers based on behavioral decision-making theory and a transtheoretical model, combined with a literature review, qualitative interviews, and two rounds of expert consultation. It includes four dimensions–behavioral decision-making motivation, behavioral decision-making influencing factors, behavioral decision-making intention, and behavioral decision-making effectiveness–with 34 items each. The scale adopts a 5-point Likert scoring method, ranging from “strongly disagree to strongly agree” with scores ranging from 1 to 5. The scores on this scale range from 34 to 170 points. Higher scores indicate better behavioral decision-making for glycemic management. In this study, the Cronbach’s alpha coefficient of the scale was 0.979, the half-half reliability was 0.919, the test–retest reliability of the total scale was 0.863, and the test–retest reliability of each dimension was between 0.717 and 0.703, indicating good reliability. At the same time, the value range of the content validity index of each item of the scale (I-CVI) is 0.780–1.000, the I-CVI of each dimension is 1.000, the content validity index of the total scale (S-CVI) is 0.828, χ^2^/df = 2.779, RMSEA = 0.080, RMR = 0.027, GFI = 0.776, NFI = 0.896, IFI = 0.931, TLI = 0.923, CFI = 0.931, PGFI = 0.656, PNFI = 0.803, and PCFI = 0.834.

#### 2.2.3 Evaluation of the independent variables

Electronic Health (E-Health) Literacy Scale. This scale was compiled by Norman et al. (36) in 2006. Guo et al. (37) translated and revised the scale into Chinese in 2013 to create a Chinese version. This scale includes three dimensions: the application ability test of online health information and services (5 items), judgment ability test (2 items), and decision-making ability test (1 item), with a total of eight items rated on a 5-point Likert scale. The score ranges from 5 to 40 points; the higher the score, the higher the level of e-health literacy. In this study, Cronbach’s alpha coefficient for this scale was 0.990.

Perceived Social Support Scale (PSSS). This scale was revised into Chinese by Jiang et al. (38) based on Zimet et al. (39). It is used to measure perceived social support. It can be divided into either two dimensions (support within the family and support outside the family) or three dimensions (support from family, friends, and others), with a total of 12 items. Using a 7-point Likert scale, strongly disagree is scored as 1 point, and strongly agree is scored as 7 points, with a total score of 12–84. The higher the total score, the higher the degree of social support perceived by the individual. In this study, Cronbach’s alpha for this scale was 0.990. This study examined two dimensions: intra-family support (four items) and extra-family support (eight items).

### 2.3 Data collection

After obtaining consent from the relevant departments of the hospital, the researchers conducted field surveys from October to November 2023 and screened pregnant women with GDM who met the inclusion criteria among outpatients and inpatients by reviewing medical records and relevant examination results. Before the investigation, the researcher first explained the purpose of the study and the relevant precautions for completed the questionnaire to the respondents and distributed the questionnaire after obtaining their informed consent. While the respondents completed the questionnaires, the investigators waited on site to answer any questions in a timely manner. After the questionnaire was completed, the investigator promptly collected it and immediately checked the completion status. If any missing or overfilled options were found, the patient was reminded to make corrections to obtain a valid questionnaire.

### 2.4 Statistical procedure

After two people screened the invalid questionnaires, SPSS 26.0, and its macro program Process 4.1 component were used to perform the corresponding statistical analysis of the data.

1. Frequency and composition comparison were used to statistically describe general information such as residence, family history of diabetes, occupation, current glycemic control methods, and history of anti-diabetic drug use.
2. The score of behavioral decision-making on glycemic management, e-health literacy, and perceived social support scale were all approximately normally distributed, so the mean ± standard deviation (M ± SD) was used to describe the variables.
3. Independent sample t-tests and one-way analysis of variance were used to compare differences in the decision-making regarding glycemic management behavior.
4. Pearson correlation analysis was used to conduct a correlation analysis on glycemic management, behavioral decision-making, e-health literacy, and understanding of social support of pregnant women with GDM.
5. Model 4 of the Process 4.1 plug-in in SPSS developed by Hayes was used to perform mediation effect testing and analysis, and the test level was α = 0.05.

### 2.5 Quality control

This study used the following measures to improve the quality of questionnaire completion: (1) Selecting the more authoritative and universal scale star online survey platform to ensure the stability of platform access; (2) The scale was presented in a scrolling manner, which is more in line with the reading habits of mobile phone users; (3) The same IP and same mobile phone could only be used to fill in the answer once, and a WeChat login was required to fill in the questionnaire to avoid repeated answers; (4) Prompts involving small gifts at the beginning and end of completing the questionnaire were used to encourage respondents to complete the questionnaire and improve the response rate of the questionnaire; (5) After questionnaire collection was completed, the researcher used the background monitoring system to delete answer sheets that had serious problems such as answering four questions in significantly less than the average response time, options being identical, to ensure data quality.

### 2.6 Ethical considerations

This research passed the hospital ethics review ([2022] Second Affiliated Hospital of Fujian Medical University Ethics Review No. (499)).

## 3 Results

### 3.1 General information on participants

As shown in Table 1, this study included 217 participants (excluding two questionnaires with illogical content, identical responses, or too short response times), of which 23.5% were rural residents. Regarding education level, participants with a bachelor’s degree or higher accounted for the highest proportion (41.5%). Employed and unemployed participants accounted for 61.3 and 38.7% of the participants, respectively. Most participants had a basic medical insurance system (80.2%).

**Table 1:** Descriptive analysis of participants’ characteristics (*N* = 217).

Variables	Variables categories	Number of people (percentage, %)	Mean score of glycemic management behavior decision (M ± SD)	t/F	*p*
Gestational weeks	<28 weeks	52 (24)	4.14 ± 0.58	1.606	0.203
	28–36 weeks	132 (60.8)	4.26 ± 0.59		
	>36 weeks	33 (15.2)	4.36 ± 0.47		
Age(years)	18–25	6 (2.8)	4.59 ± 0.52	1.846	0.140
	26–30	64 (29.5)	4.29 ± 0.58		
	31–35	97 (44.7)	4.26 ± 0.55		
	≥36	50 (23.0)	4.12 ± 0.60		
Residence	Rural	51 (23.5)	4.08 ± 0.59	0.342	<0.05
	Urban	166 (76.5)	4.29 ± 0.56		
Education	Primary school and below	9 (4.1)	3.57 ± 0.43	9.094	<0.01
	Junior school	32 (14.7)	3.99 ± 0.66		
	High school/technical secondary school	32 (14.7)	4.09 ± 0.63		
	Junior college	54 (24.9)	4.26 ± 0.50		
	College above	90 (41.5)	4.44 ± 0.49		
Family history of diabetes	Yes	44 (20.3)	4.33 ± 0.44	5.904	0.187
	No	173 (79.7)	4.22 ± 0.61		
History of gestational diabetes mellitus	Yes	61 (28.1)	4.24 ± 0.50	5.025	0.930
	No	156 (71.9)	4.24 ± 0.61		
Current methods of glycemic control	non-drug therapy	208 (95.9)	4.24 ± 0.58	0.350	0.567
	drug therapy	9 (4.1)	4.35 ± 0.54		
Occupation	Employed	133 (61.3)	4.38 ± 0.52	0.132	<0.01
	Unemployed	84 (38.7)	4.02 ± 0.59		
*Per capita* monthly household income (RMB, yuan)	≤3,000	9 (61.3)	3.59 ± 0.94	8.138	<0.01
	3,000–5,000	87 (40.1)	4.19 ± 0.56		
	≥5,000	121 (55.8)	4.33 ± 0.52		
Mode of pregnancy	Conception naturally	174 (80.2)	4.21 ± 0.58	0.149	0.141
	Human assistance	43 (19.8)	4.34 ± 0.55		
History of hypoglycemic medications	Yes	12 (5.5)	4.43 ± 0.54	0.037	0.242
	No	205 (94.5)	4.23 ± 0.58		
Medical insurance	Basic medical insurance for urban residents	174 (80.2)	4.30 ± 0.52	4.279	<0.05
	New rural basic medical insurance for rural residents	31 (14.3)	4.01 ± 0.69		
	None	12 (5.5)	4.03 ± 0.80		

### 3.2 Current situation of glycemic management behavior decision-making, e-health literacy, and perceived social support in pregnant women with GDM

Table 2 shows the scores for glycemic management behavior, decision-making, e-health literacy, and perceived social support in pregnant women with GDM. The overall mean scores of behavior decision, e-health literacy, and perceived social support are 4.24 ± 0.04, 4.05 ± 0.06, and 5.81 ± 0.07, respectively.

**Table 2:** Scores of glycemic management behavior decision-making, e-health literacy, and perceived social support in GDM pregnant women.

Variables	Items	Item mean score (M ± SD)
**Glycemic management behavior decision-making in pregnant women with GDM**	**34**	**4.24 ± 0.04**
Motivation for behavioral decision	12	4.42 ± 0.04
Influencing factors of behavioral decision	9	3.99 ± 0.05
Behavioral decision intention	8	4.29 ± 0.04
Behavioral decision effectiveness	5	4.20 ± 0.44
(**e-health) literacy**	**8**	**4.05 ± 0.06**
Application ability test	5	4.07 ± 0.59
Judging ability test	2	4.04 ± 0.59
Decision Ability Test	1	4.02 ± 0.06
**Perceived social support**	**12**	**5.81 ± 0.07**
Intra-family support	4	5.83 ± 0.08
Extra-family support	8	5.77 ± 0.07

### 3.3 Correlations among glycemic management behavior decision-making, e-health literacy, and perceived social support in pregnant women with GDM

Pearson analysis showed that the decision-making level of GDM pregnant women’s blood sugar management behavior was positively correlated with e-health literacy (*r* = 0.741, *p* < 0.01) and is positively correlated with perceived social support (*r* = 0.755, *p* < 0.01); e-health literacy and perceived social support were positively correlated (*r* = 0.694, *p* < 0.01; Table 3).

**Table 3:** Correlation analysis of glycemic management behavior decision, e-health literacy, and perceived social support in pregnant women with GDM.

Variables	Glycemic management behavioral decision making	E-health literacy	Social support
Glycemic management behavioral decision making	1	–	–
E-health literacy	0.741**	1	–
Social support	0.755**	0.694**	1

**p < 0.01.

### 3.4 The mediating effect of e-health literacy between glycemic management behavior decision-making and perceived social support in pregnant women with GDM

**Figure 1:**
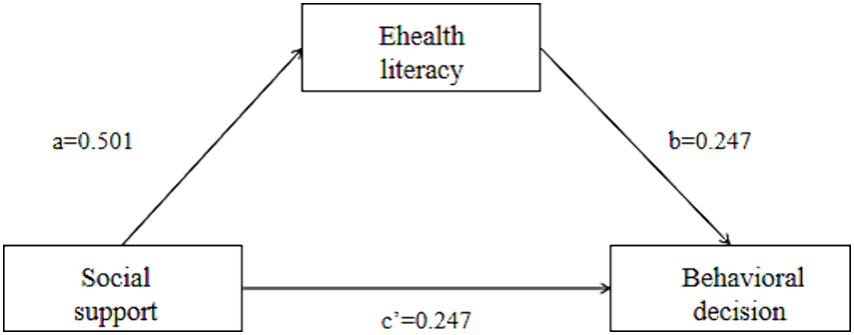
Mediation effect diagram.

Controlling for usual residence, education, occupation, *per capita* monthly household income, and type of health insurance, with glycemic management behavioral decision-making as the mean score as the dependent variable (Y), the mean score of social support for comprehension as the independent variable (X), and the mean score of e-health literacy as the mediator variable (M), Model 4 was selected; 5,000 bootstrap samples were selected, and the parameters were set up to obtain three regression path models (Figure 1). The results showed a partial mediating effect of e-health literacy between social support and glycemic management behavioral decision-making, with a mediating effect (a*b) of 0.124, a direct effect (c’) of 0.247, a total effect (mediating effect + direct effect) of 0.371, and a mediating effect to total effect ratio of 0.334. The results indicated that 33% of the effect of social support on glycemic management behavioral decision-making works through the mediating effect of e-health literacy. Further details are provided in Tables 4, 5.

**Table 4:** Mediation model tests for e-health literacy.

Regression equation (*N* = 217)		Index of fit			Significance of coefficient		
Dependent variable	Independent variable	R-sq	R	F	B	*t*	*p*
E-health literacy		0.533	0.729	39.902			<0.001
Residence					0.015	0.295	0.768
Education					0.219	3.509	<0.001
Occupation					0.000	0.007	0.994
*Per capita* monthly household income					0.007	0.152	0.879
Types of medical insurance					−0.015	−0.286	0.776
Social support					0.635	12.694	<0.001
Behavioral Decision Making for glycemic Management		0.613	0.783	55.314			<0.001
Residence					−0.021	−0.467	0.641
Education					0.166	2.924	<0.01
Occupation					−0.066	−1.189	0.236
*Per capita* monthly household income					0.009	0.186	0.852
Types of medical insurance					−0.008	−0.167	0.868
social support					0.702	15.396	<0.001
Behavioral Decision Making for glycemic Management		0.676	0.822	62.293			<0.001
Residence					−0.027	−0.639	0.523
Education					0.086	1.595	0.112
Occupation					−0.066	−1.301	0.195
*Per capita* monthly household income					0.006	0.136	0.892
Types of medical insurance					−0.003	−0.056	0.956
social support					0.467	8.418	<0.001
E-health literacy					0.369	6.402	<0.001

**Table 5:** Model effect values.

	Effect	BootSE	95%CI		Percentage of effect
			LLCI	ULCL	
Indirect effect	0.124	0.03	0.08	0.18	0.33
Direct effect	0.247	0.03	0.19	0.30	0.67
Total effect	0.371	0.02	0.33	0.41	

## 4 Discussion

This study explored the intrinsic impact mechanism of pregnant women’s perceptions of social support on their glycemic management decisions. The results showed that women’s perceptions of social support had a positive and significant impact on their glycemic management behavioral decisions, and e-health literacy indirectly increased this effect as a mediating variable. Social support has an important impact on behavioral decisions about glycemic management. In the future, the e-health literacy of pregnant women with GDM can be improved to facilitate their behavioral decision-making in glycemic management.

### 4.1 Analysis of the current situation of GDM women’s glycemic management decision making, e-health literacy, and social support

First, this study found that the average score of the decision-making items on blood sugar management behavior of pregnant women with GDM was 4.24 ± 0.04, which is higher than the theoretical median value. This is consistent with the results reported by Huang et al. (22). Most pregnant women with GDM make good decisions regarding their blood sugar management behaviors. However, Huang et al.’s study showed that as gestational age increases, the level of pregnant women’s blood sugar management behavioral decision-making decreases. However, this was not observed in the present study. This may be because the gestational age was divided differently. In the present study, the nodes at 14 and 28 weeks differed from those at 28 and 36 weeks. In this study, the behavioral decision-making motivation item had the highest average score, which may be related to the fact that this study included more pregnant women in the early stages of GDM. A meta-synthesis (40) of the experiences of pregnant women with gestational diabetes showed that pregnant women were emotionally affected by the diagnosis of GDM and developed strong coping motivation after adjustment. This study also found that pregnant women with GDM who have an urban household registration, have a high level of education, are employed, have a *per capita* monthly household income of more than 5,000 yuan, and have medical insurance have higher levels of decision-making regarding blood sugar management behavior (*p* < 0.05). This may be because pregnant women have relatively good economic status. This study showed that the factors influencing behavioral decision-making were associated with the lowest scores, indicating that pregnant women with GDM consider themselves to be the subjects and main practitioners of blood sugar management, which may be related to the conflict between traditional family concepts and the dietary control needs of GDM during pregnancy.

Then, the results of this study showed that the e-health literacy items of pregnant women with GDM have an average score of 4.05 ± 0.06, which is 3 points higher than the median value and belongs to the upper-middle level. This shows that the pregnant women with GDM in this study believe that they have the ability to use the Internet to obtain health resources. This is inconsistent with the results of previous research (41), and may be related to the higher educational level of the surveyed population in this study. In this study, pregnant women with GDM scored lower on decision-making and judgment abilities. This shows that pregnant women with GDM do not have high judgment or trust when using the Internet to obtain health information. Therefore, obtaining a large amount of health information does not necessarily mean that they can understand and differentiate between it. Previous studies (42, 43) have shown that when e-health literacy capabilities cannot meet a high level of health information needs, it will lead to negative psychological emotions and the application of incorrect health information, affecting individual health outcomes. Therefore, in the future, it may be possible to create an overall coordinated online education platform to allow women with GDM and health professionals to coordinate with each other and combine social support to address the information needs of pregnant women.

Next, the results indicated that the perceived social support scores of women with GDM were at an upper-middle level (5.81 ± 0.07), and the internal support score of the family (5.83 ± 0.08) was higher than the external support score (5.77 ± 0.07), indicating that pregnant women with GDM can obtain certain kinds of social support. Their perceptions arose from increased support within the family. Many previous studies (44–46) have also shown that social support is an important factor influencing health behaviors, from GDM maternal screening to postpartum follow-up. However, the breadth and complexity of social support determine the difficulty and long-term implementation of related intervention programs. Some studies (47, 48) have indicated that social support is a double-edged sword and does not always promote glycemic management in pregnant women with GDM. Therefore, future research should focus on the subjectivity of blood sugar management in pregnant women with GDM, while simultaneously focusing on the positive role of intra-family support. The development of information technologies such as social media may provide new avenues for this. A systematic review (49) also showed that online support interventions have the potential to improve outcomes in pregnant women with GDM.

### 4.2 Correlation analysis of glycemic management behavior decision-making, e-health literacy, and perceived social support in pregnant women with GDM

This study showed that e-health literacy in pregnant women with GDM could directly positively predict glycemic management behavior decision-making in the period (*β* = 0.741, *p* < 0.001), that is, the higher the e-health literacy of pregnant women with GDM, the higher the level of glycemic management behavior decision-making. This may be because most pregnant women with GDM lack knowledge related to glycemic management, and the development of telemedicine information technology can provide information resource support for pregnant women, improve their decision-making readiness for glycemic management, and promote healthy glycemic management behaviors. A scoping review (50) also indicated that mobile health and telemedicine can be effective platforms for improving glycemic management in women with GDM; however, scientific information support is the basis for effective glycemic management. Liu et al. (51) showed that although there is no direct relationship between e-health activities and health behaviors–that is, e-health activities do not necessarily translate into healthy life behaviors–and the influencing factors are complex, the influence of e-health information on individual health attitudes and decisions is beyond doubt. Therefore, in the future, improving the reading ability of pregnant women with GDM can be considered to help them directly access electronic health resources, as well as improve their ability to use information technology, and their decision-making ability to obtain information to make correct choices, to help them better implement glycemic management. In addition, studies (52) have shown that e-health literacy can support medical care by providing greater efficiency, improving health outcomes, and reducing the cost of medical services and has the potential to support challenging long-term health behaviors and provide effective support for pregnant women with GDM with low education, living in remote areas, and low economic income. At the same time, it also provides a new solution for changing the persistence of glycemic management behavior of pregnant women with GDM.

This study showed that the perceived social support of pregnant women with GDM was positively correlated with glycemic management behavior decision-making (*β* = 0.755, *p* < 0.001), that is, a high level of perceived social support can improve the negative psychological state of pregnant women with GDM when facing the disease diagnosis, and provide material support to help them avoid negative decision-making and improve their compliance with glycemic management behavior. Wah et al. (48) found that pregnant women with GDM who received adequate social support were better able to make scientific glycemic management decisions, among which family support was one of the most easily available. According to the social ecological model (53), in addition to the internal factors of individuals, multilevel external sociological factors should be considered that affect disease self-management behavior, including self-regulation, family, professional medical staff, community, work, policy, and other factors, among which family support is an important factor. Therefore, future intervention studies should consider the factors influencing glycemic management behavior decision-making in pregnant women with GDM in many respects, taking family support as the entry point, such as letting patients and family members participate in glycemic management decision-making, focusing on understanding the unique information needs of family members, helping pregnant women break through the decision-making dilemma, and establishing a stable glycemic management support system.

### 4.3 Analysis of the mediating effect of e-health literacy between glycemic management behavior decision-making and perceived social support in pregnant women with GDM

This study found that the total effect of perceived social support in predicting glycemic management behavior decision making was significant. When e-health literacy was introduced, perceived social support could still positively predict glycemic management behavior decision-making (*β* = 0.280, *p* < 0.001), indicating that perceived social support could not only directly affect the glycemic management behavior decision-making of pregnant women with GDM, it could also have an indirect effect through e-health literacy, which accounted for 38% of the total effect, indicating that e-health literacy is an important way for pregnant women with GDM to improve glycemic management behavior decision-making through perceived social support. Studies have shown (54, 55) that the effectiveness of early self-management education for women with GDM can only be maintained for 3 months without effective support strategies. Effective social support systems combined with telemedicine platforms, such as Internet platforms and telephone follow-ups, can promote the maintenance of glycemic management behavior in women with GDM. On the one hand, parents and peers in the social network can provide explicit or implicit emotional support for pregnant women with GDM, which can help alleviate their physical and psychological problems. At the same time, the support information provided by members of the same disease patient and other members also strengthens individual disease awareness and ultimately promotes scientific behavioral decision-making. On the other hand, social support may indirectly affect the glycemic management decision-making behaviors of pregnant women with GDM through the intermediate variable of e-health literacy. The comprehensive use model of e-health (31) points out that individuals with good social support tend to use the Internet and electronic products to learn about health problems but can also make health choices that are beneficial to their own health by comparing and evaluating the online health information obtained. Hence, social support is helpful to improve e-health literacy, and improvement of e-health literacy can promote scientific decision-making regarding glycemic management of pregnant women with GDM. Another review (56) suggested that computers and web-based health information technologies can address day-to-day tasks and monitoring required for disease self-management without the limitations of time and geography, which frees social support providers (such as health professionals) to provide highly personalized support for more complex problems.

### 4.4 Limitations

Our study has some limitations. (1) This was a cross-sectional study and could not confirm the causal relationship between perceived social support, e-health literacy, and glycemic management behavior decisions. Longitudinal studies or intervention studies can be used to verify the results of this study in the future. (2) The e-health literacy scale is universal and does not consider the specificity of GDM in pregnant women, which may have affected the results. (3) The sample size of this study was relatively limited, and future studies should expand the sample size to further verify these results.

## 5 Conclusion

In conclusion, the results of this study suggest that social support may indirectly affect glucose management behavioral decisions in pregnant women with GDM by affecting their e-health literacy. Considering that social support is difficult to change in a short period, this study suggests that professional medical staff should pay attention to the improvement of e-health literacy of pregnant women with GDM and improve women’s e-health literacy by opening relevant courses and popularizing official scientific health information websites to improve scientific decision-making regarding glycemic management behavior.

## Data availability statement

The original contributions presented in the study are included in the article/supplementary material, further inquiries can be directed to the corresponding author.

## Ethics statement

The studies involving humans were approved by Second Affiliated Hospital of Fujian Medical University Ethics Review. The studies were conducted in accordance with the local legislation and institutional requirements. Written informed consent for participation was not required from the participants or the participants' legal guardians/next of kin because this study was an online survey, and verbal consent was obtained from the respondents prior to the survey. Written informed consent was obtained from the individual(s) for the publication of any potentially identifiable images or data included in this article.

## Author contributions

PY: Writing – original draft. KH: Writing – review & editing. SL: Writing – review & editing. ZX: Writing – review & editing, Methodology. ZM: Writing – review & editing, Investigation, Resources. XY: Supervision, Writing – review & editing. ZH: Writing – review & editing.
