# Erratum: The mediating effect of e-health literacy on social support and behavioral decision-making on glycemic management in pregnant women with gestational diabetes: a cross-sectional study

**DOI:** 10.3389/fpubh.2024.1477643

**Published:** 2024-08-20

**Authors:** Frontiers Production Office

**Affiliations:** Frontiers Media SA, Lausanne, Switzerland

**Keywords:** gestational diabetes, pregnancy, glycemic management, behavioral decision making, health behavior, social support

Due to a production error, the first two authors Peng Yumei and Ke Huiying were not shown to share first authorship.

A correction was made to the author list to signify this, and the following note was inserted:

“These authors share first authorship.”

The publisher apologizes for this mistake. The original version of this article has been updated.

